# Efficacy and complications of blocking screws fixation in the treatment of lower limb long bone fracture: a meta-analysis

**DOI:** 10.3389/fsurg.2025.1560150

**Published:** 2025-03-21

**Authors:** Zhaoguo Jin, Ding Wang

**Affiliations:** Department of Orthopedics, First People’s Hospital of Linping District, Hangzhou, China

**Keywords:** blocking screws, intramedullary nail, tibia, femur, fractures, fixation, meta-analysis

## Abstract

**Background:**

Long bone fractures, especially in the lower limbs, are highly prevalent in orthopedic practice. These fractures can significantly impair patients' mobility and quality of life. Intramedullary nails are a mainstay treatment, offering reliable fracture fixation. However, the addition of blocking screws has introduced an element of uncertainty regarding surgical outcomes. This meta-analysis evaluated the efficacy and complications of blocking screw fixation for lower limb long bone fractures (LLLBF).

**Methods:**

A comprehensive and systematic search was conducted across eight databases, namely the Cochrane Library, PubMed, EMbase, Web of Science, CNKI, China Biomedical Literature Database (CBM), VIP, and WanFang, to identify relevant controlled trials. Before data analysis, the quality of each study was rigorously assessed. Subsequently, the data were analyzed using the Review Manager 5.3 (RevMan 5.3) software to ensure a reliable and accurate synthesis of the evidence.

**Results:**

A total of 15 studies were incorporated into the analysis. Compared with the control group, the experimental group demonstrated a significantly shorter fracture healing time (standardized mean difference, SMD = −2.18; 95% confidence interval, CI: −3.17 to −1.20; *P* < 0.001), suggesting a substantial effect in favor of the intervention. Additionally, the experimental group had a longer operation time (SMD = 15.81, 95% CI: 4.28, 27.34, *P* = 0.007), less intraoperative bleeding (SMD = −75.60, 95% CI: −127.93, −23.27, *P* = 0.005), and fewer complications (odds ratio, OR = 0.51, 95% CI: 0.31, 0.84, *P* = 0.008). However, no significant difference was observed in the fracture healing rates between the two groups (OR = 1.09, 95% CI: 0.98, 1.20, *P* = 0.098).

**Conclusion:**

The findings of this study suggest that the use of intramedullary nails in conjunction with blocking screws could potentially be an effective treatment option for patients with lower limb long bone fractures. However, to confirm this efficacy, additional high - quality research, preferably well-designed randomized controlled trials with large sample sizes and long-term follow - up, is warranted.

## 1 Introduction

With the incidence of accidents such as car crashes and heavy-object-related injuries has surged. These are predominantly caused by high - energy traumas, which present formidable challenges in the treatment of complex fractures (1, 2). Long bone fractures constitute approximately 4% of emergency trauma cases. Among them, lower limb fractures, especially those involving the femoral and tibiofibular shafts, are highly prevalent (3, 4). A femoral shaft fracture spans from the lesser trochanter to the adductor tubercle, while the tibial shaft is located below 6 cm of the tibial plateau and above 5 cm from the ankle joint (5, 25). Notably, around 13.7% of all fractures involve long bones. Tibial shaft fractures are particularly common, mainly affecting men aged 20–50 years, with a male - to - female ratio of approximately 2.8:1 (6, 24).

Conventional treatment modalities for femoral, tibial shaft, and metaphyseal fractures include the application of steel plates, external fixators, and bone traction. However, intramedullary nailing has emerged as a superior option due to its higher fracture - healing efficiency and fewer complications (3, 6). By minimizing the risk of delayed union or nonunion, it safeguards the crucial blood supply to the fracture site. As a central internal fixation technique, the intramedullary nail adheres to biomechanical principles (23). It directly contacts the bone tissue within the medullary cavity, effectively stabilizing the fracture ends and restricting their movement (7). This not only optimizes the healing process but also improves postoperative outcomes. The internal splint - like structure of the intramedullary nail, formed through its direct contact with the inner wall of the bone, stabilizes the fracture by transmitting forces and limiting displacement. Its axial sleeve structure, in combination with the bone in the medullary cavity, provides robust fixation, especially in comminuted fractures where the fragment size exceeds the radius of the nail. By restoring the long bone's axial force line and evenly distributing stress, the intramedullary nail mitigates the risks of stress shielding and refracture. The closed - reduction and nailing technique, performed at a distance from the fracture site, preserves the periosteal blood supply and the osteogenic growth factors in the fracture hematoma. This, in turn, reduces complications and promotes early functional recovery, highlighting the advantages of intramedullary nailing over traditional treatment methods (3, 6).

Traditional treatments for femoral, tibial shaft, and metaphyseal fractures, such as the use of steel plates, external fixators, and bone traction, each have their specific indications and limitations. For example, steel plates are typically employed for fractures that demand anatomical reduction and rigid fixation. Nevertheless, they often involve extensive soft - tissue dissection, which can compromise the blood supply and elevate the risk of infection (3, 6). External fixators are valuable in cases of open fractures or when immediate stability is required in the presence of soft-tissue injuries. However, they can be inconvenient for patients and are associated with a higher risk of pin - tract infections.

Intramedullary nailing, on the other hand, is preferred in several situations. It is particularly suitable for long bone fractures, such as those of the femoral and tibial shafts. This is because it offers several advantages over alternative fixation methods. Firstly, it has a higher healing efficiency and reduces the risk of delayed healing or nonunion. By minimizing the risk of these complications, it preserves vital blood flow to the fracture site. As a central internal fixation method, the intramedullary nail aligns with biomechanical principles. It directly contacts the bone tissue inside the medullary cavity, stabilizing the fracture ends and limiting their motion. This approach optimizes healing and enhances postoperative outcomes. Secondly, the internal splint - like structure of the intramedullary nail, which is formed by its direct contact with the bone's inner wall, stabilizes the fracture by transmitting force and limiting movement. Its axial sleeve structure, formed with the medullary cavity bone, ensures strong fixation, especially in comminuted fractures where fragment size surpasses the nail's radius. This helps in restoring the long bone's axial force line and evenly distributing stress, minimizing stress shielding and refracture risks.

Moreover, the technique of closed reduction and nailing, which is often performed distant from the fracture site, preserves periosteal blood supply and the osteogenic growth factors in the fracture hematoma. This not only lowers the risk of complications but also promotes early functional recovery. In summary, intramedullary nailing is a preferred option when aiming to achieve efficient fracture healing, maintain stability, and reduce the risk of complications, especially in lower limb long bone fractures.

## 2 Materials and methods

### 2.1 Selection of studies

Design of the Investigation: Only published randomized controlled trials (RCTs) on the effectiveness of blocking screws fixing in the management of long bone fractures in the lower leg were included. This was to ensure the reliability and validity of the study, as RCTs are considered the gold standard for evaluating treatment effects in a superiority study. Animal experimentation was excluded.

### 2.2 Fracture healing rate

During a localized examination, the doctor will check the injured area for pressure, tenderness, or unusual activity. If the fracture site is pain - free and there is no discomfort upon palpation or application of light pressure, it may suggest that the fracture is healing. For functional testing: In the case of the upper extremity, the patient may be asked to lift a 1-kg weight and hold it for 1 min to test arm stability. For the lower extremity, the patient may be required to walk a certain distance (either 3 min or 30 steps) to assess weight - bearing ability and gait. Before performing any functional test, the physician will conduct a safety review to ensure that the test will not cause the fracture to re - displace or deform.

Radiographic methods play a crucial role in assessing fracture healing. X-rays are commonly used to observe if the fracture line is blurred and if a bone scab forms and connects the two ends of the fracture. This is done by comparing consecutive radiographs taken after the fracture. The presence of a bone scab and the disappearance of the fracture line are signs that healing is in progress or has been completed. Additionally, a CT scan can offer more detailed images, which are useful for determining the extent of fracture healing.

Finally, the doctor records the patient's recovery progress, tracking healing time points and comparing them to standard healing times. In studies or statistical reports, fracture healing rates are typically calculated as the number of healed cases over a specific period divided by the total number of cases.

### 2.3 Identification of individuals

Individuals with lower limb long bone fractures (LLLBF) were considered for inclusion. The fractures included those of the femoral shaft (extending from the lesser trochanter to the adductor tubercle) and tibial shaft (lying below 6 cm of the tibial plateau and above 5 cm from the ankle joint).

### 2.4 Interventions criteria

Intervention group: Patients with LLLBF were treated with intramedullary nails and blocking screws.

Control group: Patients with LLLBF received a single intramedullary nail or intramedullary nail combined with a steel plate.

### 2.5 Inclusion and exclusion criteria

Inclusion Criteria:

(1) Patients of all ages were included, as long as they had a confirmed LLLBF. However, special attention was paid to studies that reported data separately for different age groups, such as pediatric patients (aged 0–18 years), young adults (19–40 years), middle - aged adults (41–60 years), and elderly patients (above 60 years).
(2) Both simple fractures (e.g., transverse, oblique fractures) and comminuted fractures were included. Fractures were classified based on standard radiological criteria.
(3) Patients with various comorbidities, such as diabetes mellitus, hypertension, cardiovascular diseases, and respiratory diseases, were included. Studies that reported the impact of comorbidities on treatment outcomes were especially noted.
(4) Patients who had no prior treatment for the index fracture or those who had received non - definitive treatments (e.g., temporary splinting) were included.

Exclusion Criteria:

(1) Patients with pathological fractures caused by conditions like bone tumors or osteoporosis - related fractures were excluded.
(2) Those who had received definitive treatment for the fracture before the study (such as external fixation for a long - term period or open reduction and internal fixation with other implants) were excluded.
(3) Studies that did not clearly report the relevant patient characteristics (age, fracture type, comorbidities, prior treatments) were excluded from the analysis.

**Figure 1:**
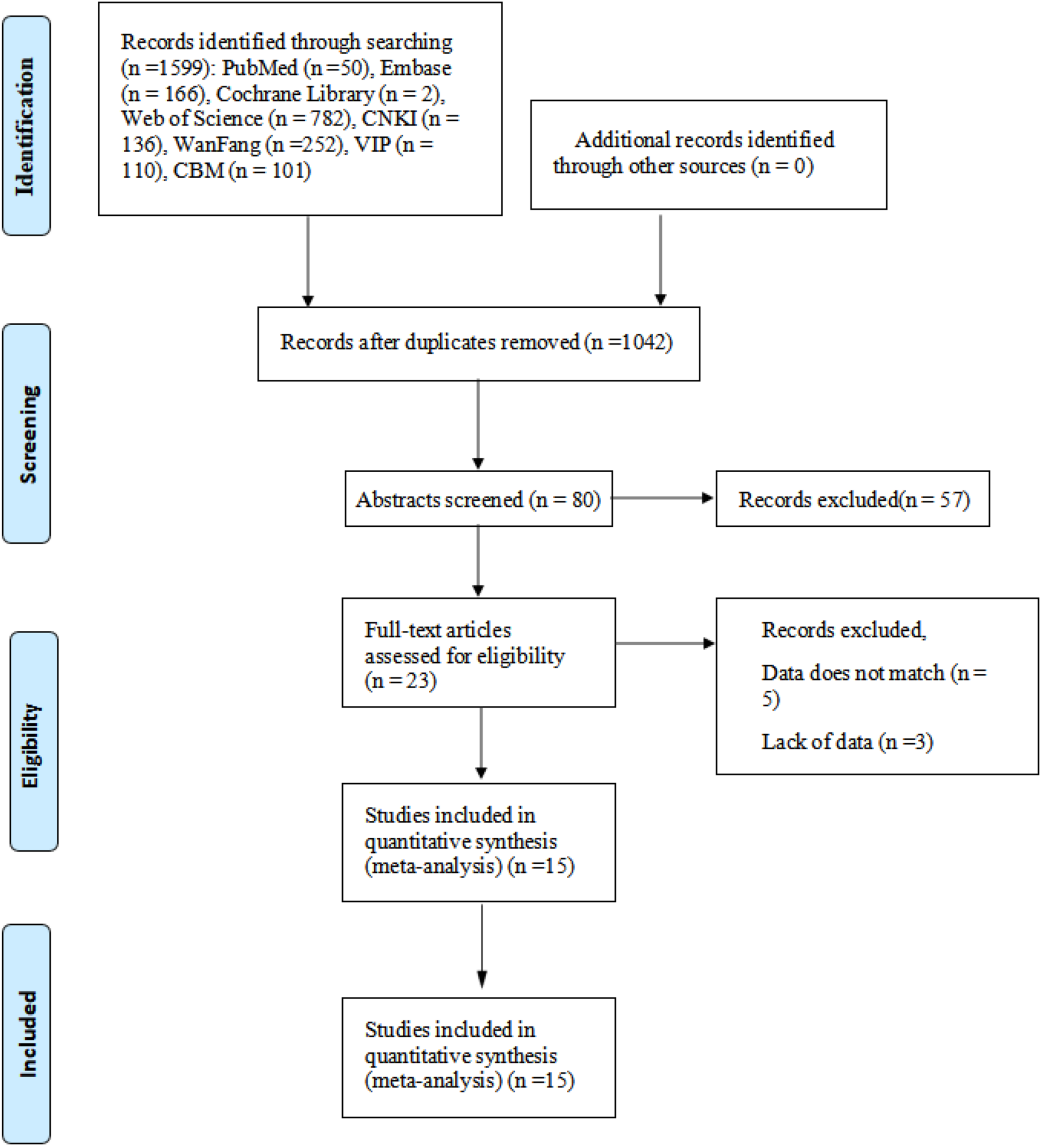
Flow chart.

The detailed study selection process is outlined in Figure 1.

### 2.6 Evaluating the outcomes

Outcome indicators for individuals with LLLBF; According to research, the assessment tools for the effects of blocking screws fixation to treat LLLBF are: (1) Fracture healing time; (2) Fracture healing rate; (3) Operation time; (4) Intraoperative bleeding; (5) Complications. The literature included in this study evaluated outcome measures using at least one of the above scales.

### 2.7 Search strategies

The Cochrane Library, PubMed, EMbase, Web of Science, CNKI, China Biomedical Literature Database (CBM), VIP, and WanFang are among the online databases that the machine accesses. The search term is “blocking screws”, “poller screws” and “fracture”. The search period lasted from the library's founding to February 2022. The precise procedures for investigating literature are as follows: (1) look for appropriate articles in databases in both Chinese and English; examine the title of the paper, the abstract, and significant keywords to determine the search phrases for the present investigation; (2) The “MeSH Terms” were utilized in the English repository search, which combined topic words and keywords to find the subject concepts (22).

### 2.8 Retrieval of data and quality evaluation

The paper filtering method was carried out independently by 2 investigators following a preliminary review of the summary. The findings of the article filtering were subsequently gathered by studying the whole content. Until the outcomes are unanimous, researchers can compare evaluation findings, discuss opposing literary works, or contact a third investigator. Basic knowledge regarding the research, research type, investigation object, number of participants, intervention content, outcome measures, etc., are among the data that were retrieved.

### 2.9 Computational analysis of the data

Review Manager (RevMan) was utilized to carry out this computational analysis. Effects are combined: This study's outcome measures were all measurable data, including its various evaluation techniques. The standardized mean difference (SMD) and 95% confidence interval (CI) were employed as a measure of effect due to significant variations in the values. Chi-square tests were used to assess heterogeneity between studies. If *P* > 0.1 and *I*^2^ < 50%, a fixed-effects model was applied; if *P* < 0.1 and *I*^2^ ≥ 50%, a random-effects model was used. To explore the sources of heterogeneity, subgroup analyses were conducted based on potential confounding factors such as fracture location (femoral fractures vs. tibial fractures) and surgical methods (different types of intramedullary nail fixation and whether combined with steel plates).

To assess bias, we used the Cochrane Risk of Bias tool for RCTs. This tool evaluates bias across multiple domains, including random sequence generation, allocation concealment, blinding of participants and personnel, blinding of outcome assessment, incomplete outcome data, selective reporting, and other sources of bias. Each study was carefully evaluated in these aspects, and the results of the bias assessment were used to inform the interpretation of the study findings.

## 3 Results

### 3.1 Selection of Key studies and overview of overall research

A systematic review and meta-analysis of 15 studies involving 480 patients in the experimental group and 570 in the control group assessed the effectiveness of intramedullary nails augmented with blocking screws for Lower Limb Long Bone Fractures (LLLBFs) (Table 1). Results showed a significantly shorter fracture healing time in the experimental group (SMD: −2.18; 95% CI: −3.17, −1.20; *P* < 0.001). However, there was no statistically significant difference in fracture healing rates between the groups (OR: 1.09; 95% CI: 0.98, 1.20; *P* = 0.098) (7, 8, 10, 12, 14). Despite this, the experimental group experienced longer operation times (SMD: 15.81; 95% CI: 4.28, 27.34; *P* = 0.007) but had significantly less intraoperative bleeding (SMD: −75.60; 95% CI: −127.93, −23.27; *P* = 0.005) and fewer complications (OR: 0.51; 95% CI: 0.31, 0.84; *P* = 0.008) compared to the control group.

**Table 1:** The key features of the research that are incorporated.

Study (ref.)	Sample Size (T/C)	Man/Woman	Age (years) (Mean ± SD）(T/C)	T/C	Main Outcomes
Song (7)	23/26	30/19	39.2 ± 11.8/39.8 ± 12.5	BS/NBS	①②③⑤
Peat et al. (8)	66/88	92/62	41.3 ± 17.7	BS/NBS	②⑤
Fawdington et al. (9)	10/20	15/15	39/40	BS/NBS	①
Van Dyke et al. (10)	46/70	69/47	36.6 ± 15.1/39.9 ± 17.7	BS/NBS	①②
Schumaier et al. (11)	30/54	60/24	43 ± 18/41 ± 19	BS/NBS	⑤
Guo et al. (12)	33/63	68/28	43.9 ± 16.9/50.2 ± 19.3	BS/NBS	①②③④⑤
Guo et al. (13)	17/19	21/15	36.9 ± 12.8/34.1 ± 15.3	BS/NBS	①③
Mao et al. (14)	24/29	32/21	45.91 ± 15.32/43.63 ± 18.13	BS/NBS	①②③④⑤
Bai (15)	35/35	47/23	39.05 ± 6.03/38.25 ± 6.16	BS/NBS	①
Li et al. (16)	17/19	21/15	36.90 ± 12.80/34.10 ± 15.30	BS/NBS	①②③④⑤
Wei et al. (17)	53/53	58/48	37. 39 ± 5. 44/38. 68 ± 5. 29	BS/NBS	①③④⑤
Pan et al. (18)	31/24	40/15	39. 3/37. 8	BS/NBS	①③
Deng et al. (19)	43/44	54/33	43.02 ± 7.68/42.82 ± 7.63	BS/NBS	①③④
Meng et al. (20)	25/23	31/17	38.73 ± 12.85/36.85 ± 12.77	BS/NBS	①③④
Wang et al. (21)	27/27	35/19	42.7 ± 4.9/43.1 ± 6.2	BS/NBS	③④⑤

T/C: trial group/control group. BS, Blocking screws; NBS, Non-Blocking screws. ① Fracture healing time; ② Fracture healing rate; ③ Operation time; ④ Intraoperative bleeding; ⑤ Complications.

### 3.2 Fracture healing time

**Figure 2:**
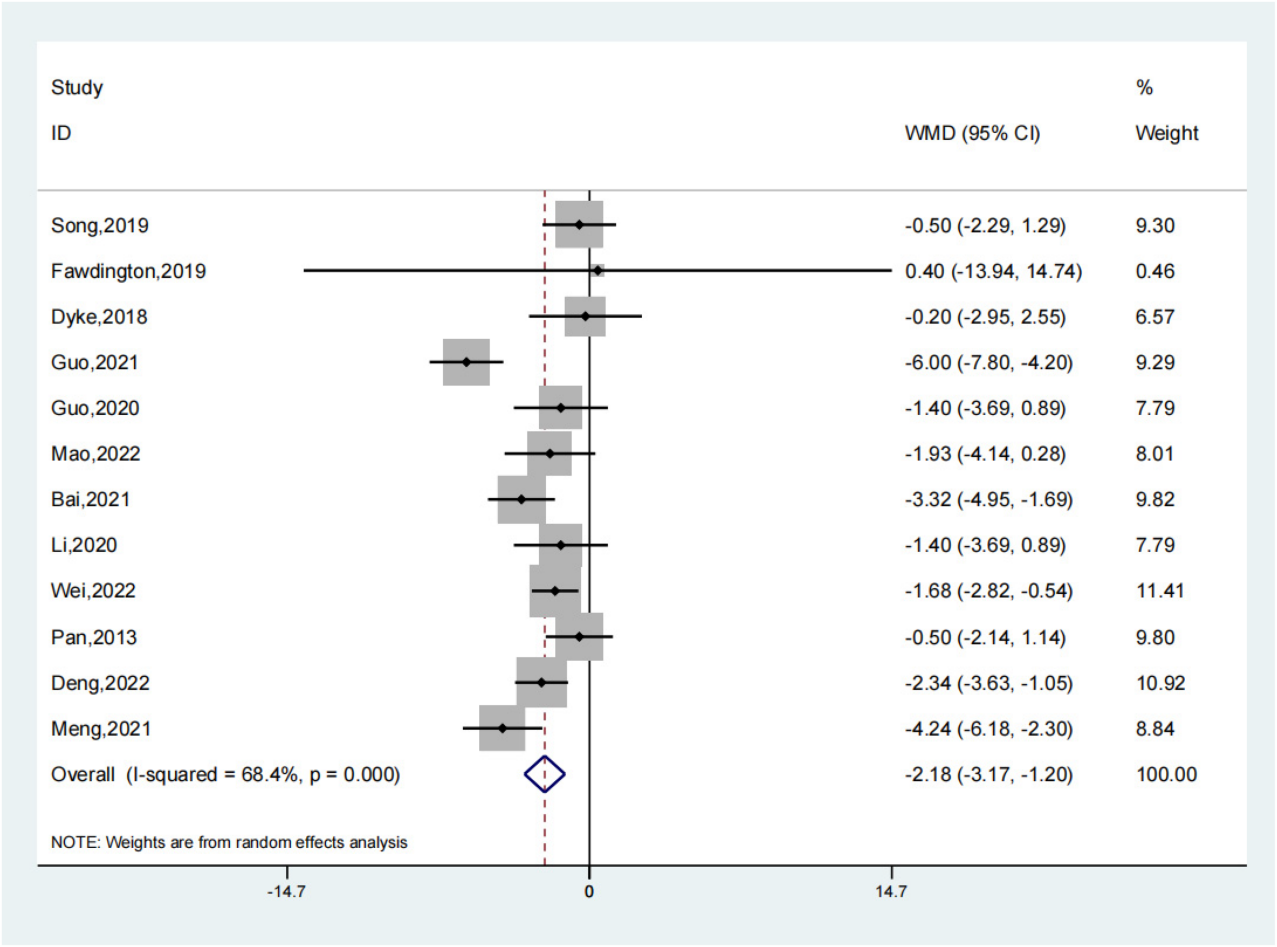
Fracture healing time depiction in the forest plot.

**Figure 3:**
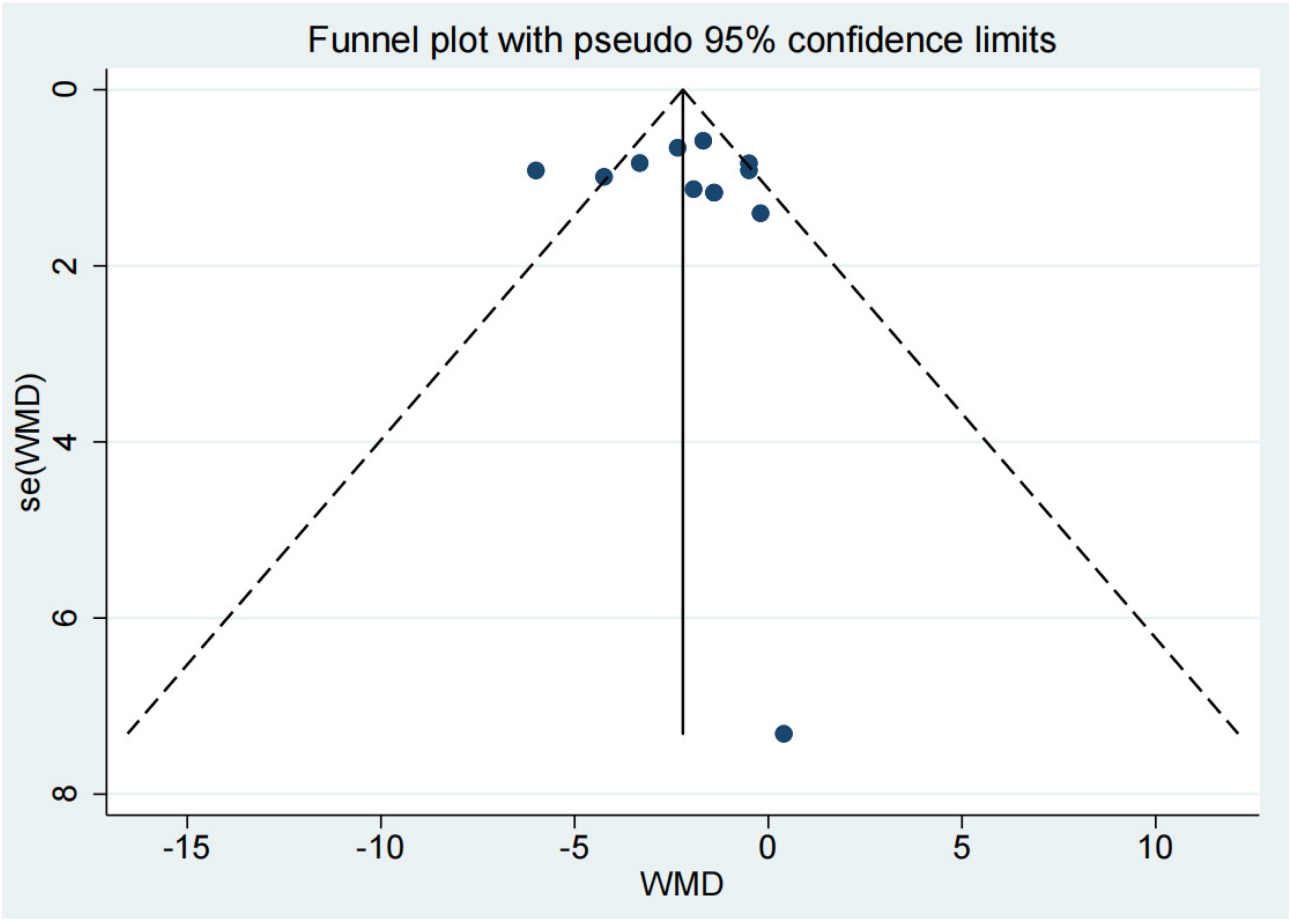
The fracture healing time is depicted in a funnel plot.

**Figure 4:**
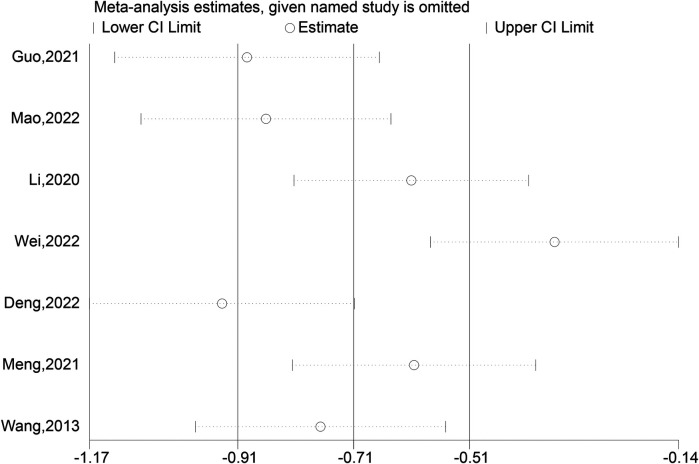
Sensitivity analysis of the fracture healing time.

The meta-analysis of 15 studies indicated that using intramedullary nails with blocking screws for LLLBFs led to a marked decrease in fracture healing time. Based on a synthesis of 12 studies (7, 9, 10, 12–17, 19–21), the fracture healing period was demonstrably shorter for the group treated with intramedullary nails and blocking screws vs. the control group (SMD: −2.18; 95% CI: −3.17, −1.20; *P* < 0.001). The analysis of fracture healing time across studies is presented in a forest plot, illustrating the comparative outcomes between treatment groups (Figure 2). A funnel plot analysis indicated a symmetric distribution, suggesting low publication bias (Figure 3). A sensitivity assessment, conducted given the observed outcome heterogeneity, validated the stability of the results. Intramedullary nailing combined with blocking screws notably expedited recovery in Lower Limb Long Bone Fractures (LLLBF) patients. Both Begg's Test (0.891) and Egger's Test (0.21, Figure 4) confirmed the absence of substantial publication bias, strengthening the study's conclusions.

Overall, 15 studies were included in this meta - analysis to comprehensively evaluate the efficacy and complications of blocking screw fixation for lower limb long bone fractures. However, when specifically analyzing fracture healing time, only 12 studies were considered. This is because 3 studies did not report complete or relevant data regarding fracture healing time. We strictly adhered to our data inclusion criteria, which required accurate and comparable data on fracture healing time for inclusion in this particular analysis. By excluding these 3 studies, we aimed to ensure the reliability and validity of the results on fracture healing time, minimizing potential biases that could arise from incomplete or inconsistent data.

In summary, the integration of 12 studies highlights that the use of intramedullary nails and blocking screws in LLLBF treatment leads to a substantial reduction in fracture healing time, with consistent findings and minimal evidence of publication bias. This intervention thus demonstrates superior efficacy in accelerating the healing process.

### 3.3 Fracture healing rate

**Figure 5:**
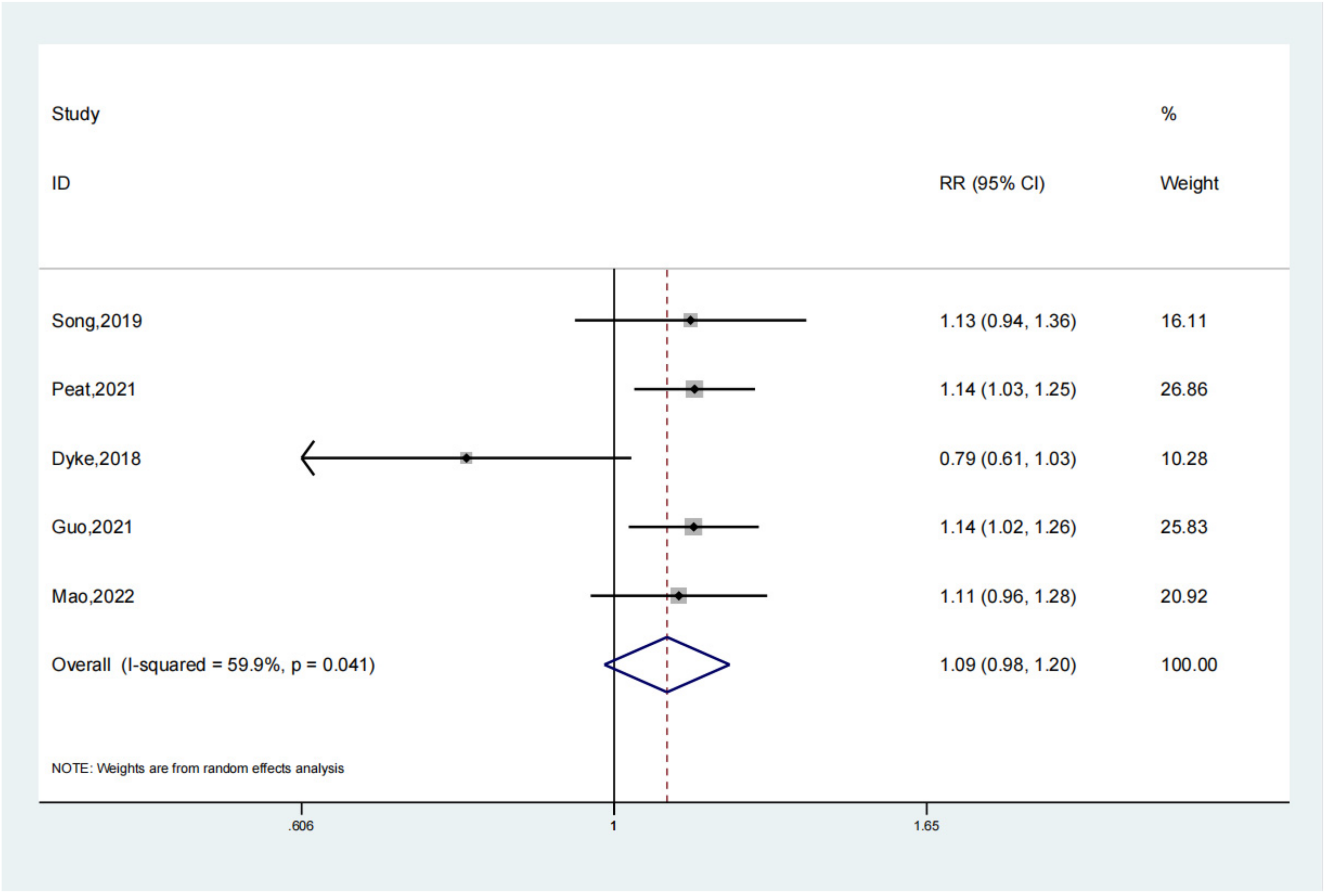
Fracture healing rate depiction in the forest plot.

**Figure 6:**
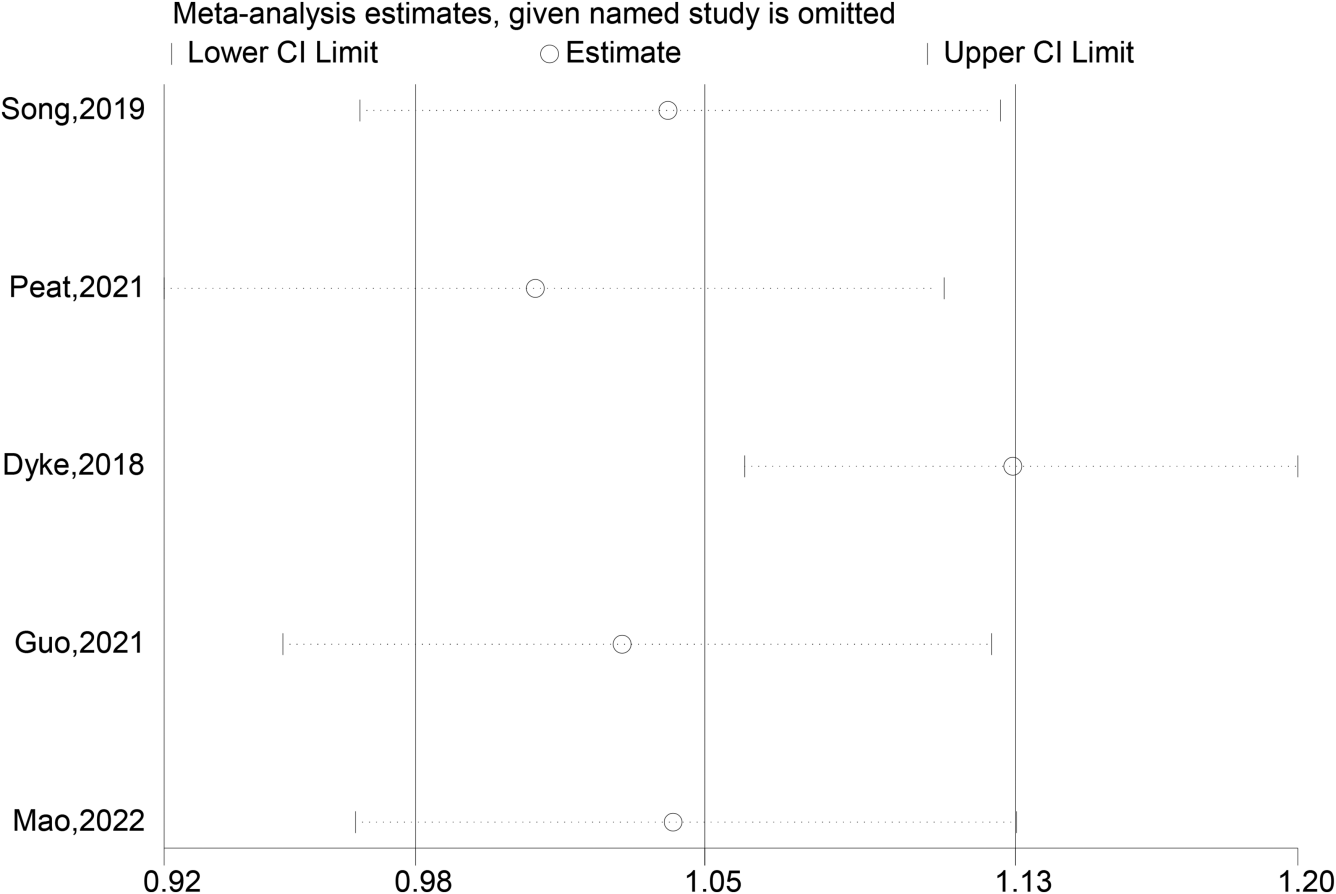
Sensitivity analysis of the fracture healing rate.

Our study clearly shows that there was no statistically significant difference in fracture healing rates between the experimental and control groups (OR: 1.09; 95% CI: 0.98, 1.20; *P* = 0.098). Although the odds ratio of 1.09 is close to the upper limit of the confidence interval (7, 8, 10, 12, 14), indicating a possible trend in favor of the experimental group (intramedullary nails combined with blocking screws), this did not reach the traditional level of statistical significance (Figure 5). In clinical practice, even a non - significant difference in treatment methods that might affect the fracture healing rate can be important for individual patients. For instance, in a large - scale clinical scenario, a small increase in the fracture healing rate could lead to better outcomes for a substantial number of patients. However, based on our current study, we cannot conclude that the combination of intramedullary nails and blocking screws has a significant impact on fracture healing rates. The close - to - significant result may be attributed to the relatively small sample size in our study. Therefore, future research with larger sample sizes or more optimized study designs is required to accurately assess the effect of intramedullary nails combined with blocking screws on fracture healing rates and provide more reliable evidence for clinical practice (Figure 6).

### 3.4 Operation time

**Figure 7:**
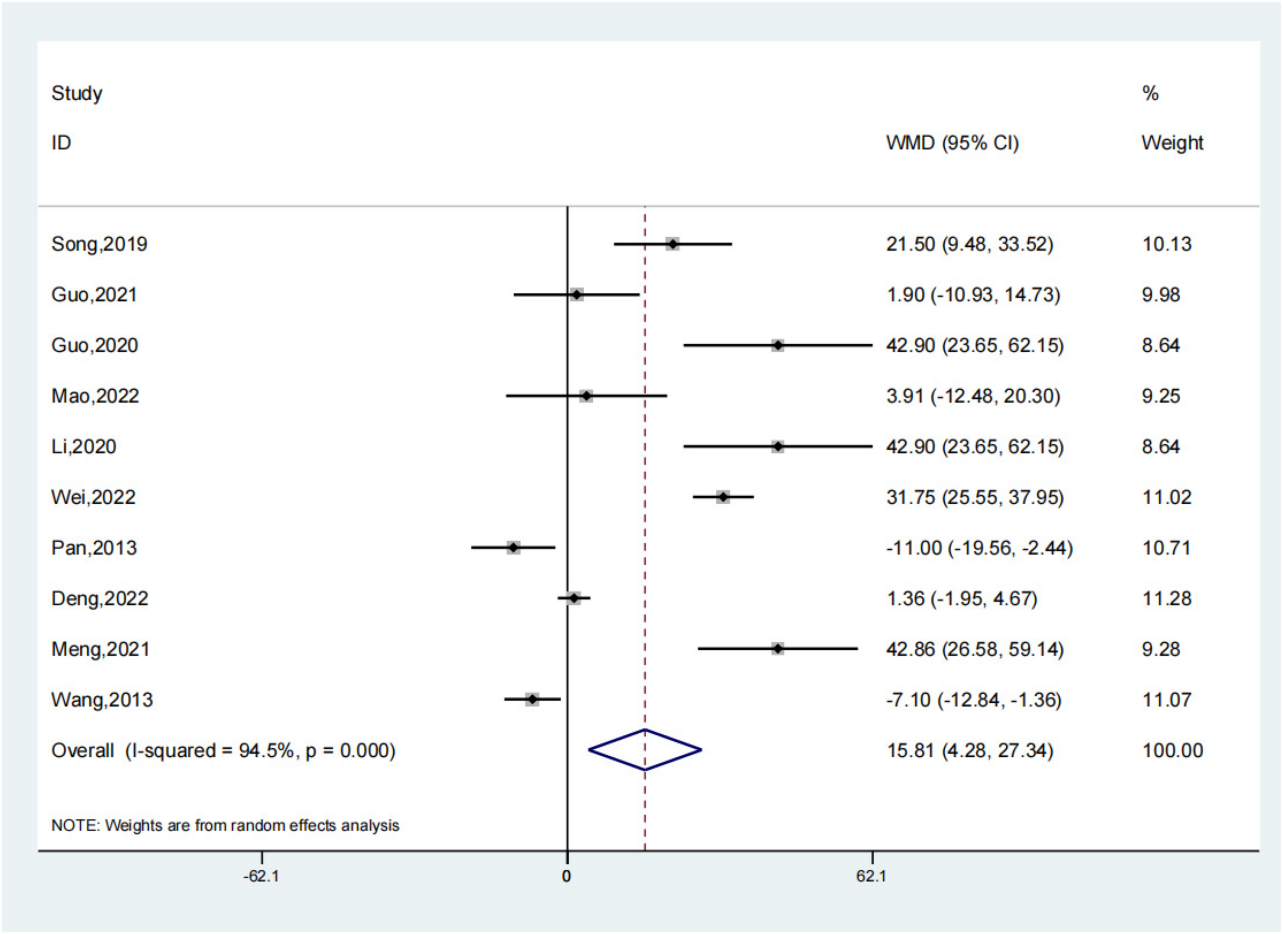
Operation time depiction in the forest plot.

**Figure 8:**
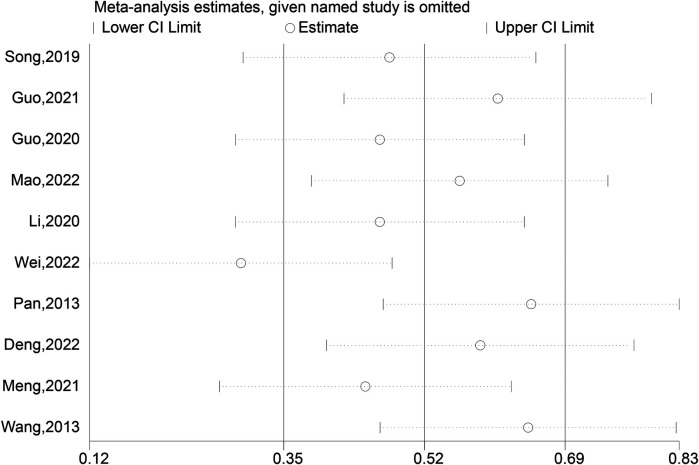
Sensitivity analysis of the operation time.

Ten meticulously (3, 7, 12–14, 16, 17, 19–21) conducted trials were analyzed to compare the operation times between the experimental group, which employed intramedullary nails and blocking screws, and the control group. This comprehensive analysis disclosed a statistically noteworthy prolongation of the surgical duration in the experimental group relative to the control group (SMD: 15.81; 95% CI: 4.28, 27.34; *P* = 0.007, Figure 7). The adoption of the novel technique involving intramedullary nails and blocking screws for LLLBF treatment resulted in a more extended operation time, potentially impacting the overall surgical workflow. To account for the observed variability in the outcomes across these trials, a sensitivity analysis was carried out, revealing a substantial degree of heterogeneity (Figure 8), which underscores the need for a deeper understanding of the factors contributing to these differences.

### 3.5 Intraoperative bleeding

**Figure 9:**
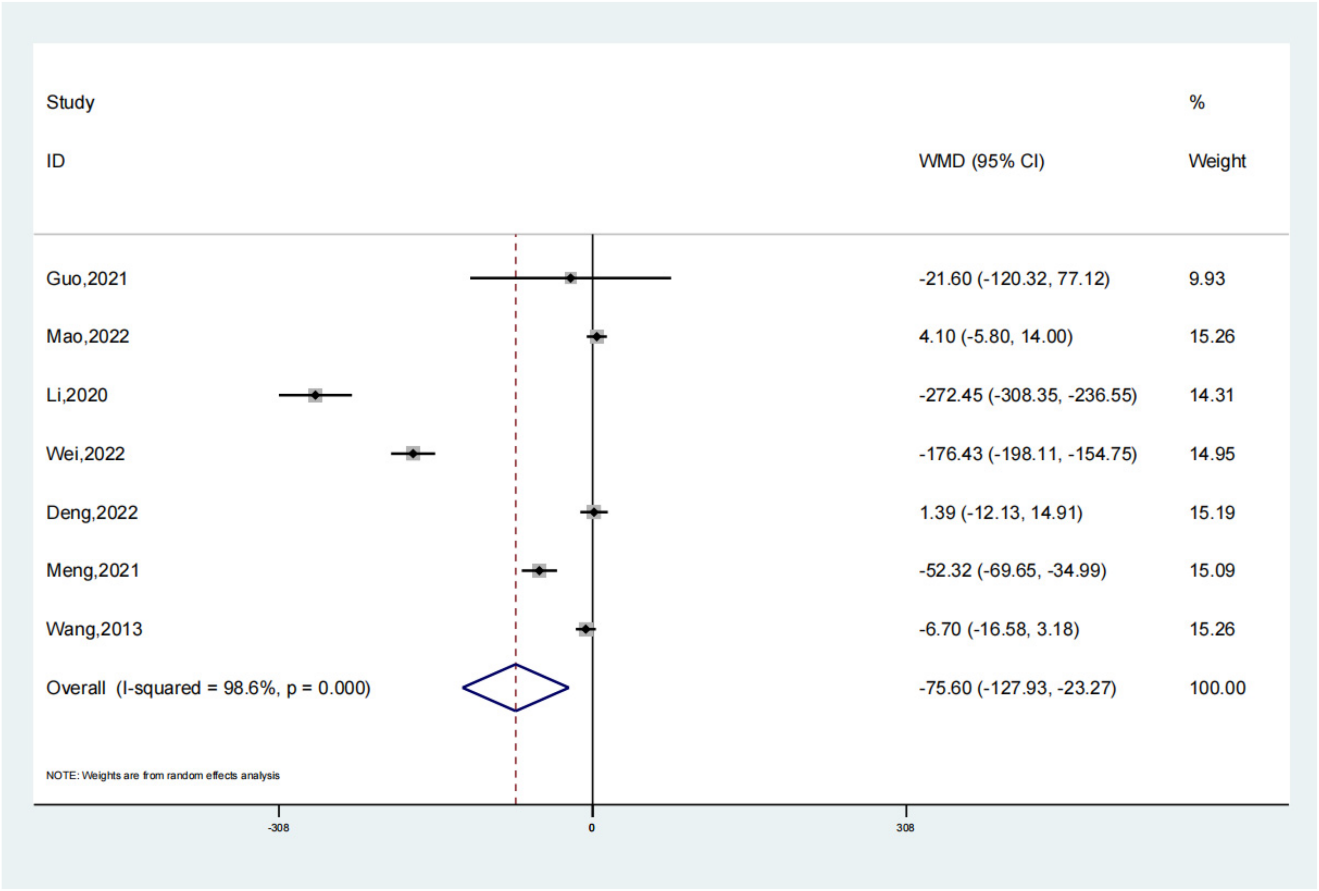
Intraoperative bleeding depiction in the forest plot.

**Figure 10:**
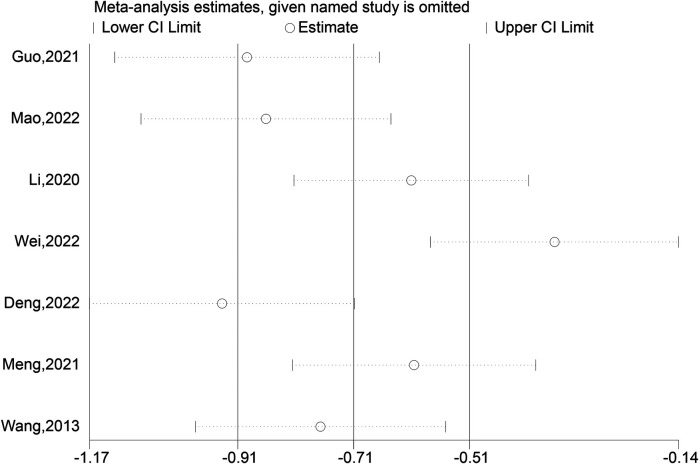
Sensitivity analysis of the intraoperative bleeding.

Seven comparative studies were reviewed to assess intraoperative bleeding in both the experimental group, utilizing intramedullary nails and blocking screws, and the control group. The data conclusively demonstrated a considerable decrease in blood loss during surgery for the experimental group as compared to the control (SMD: −75.60; 95% CI: −127.93, −23.27; *P* = 0.005, Figure 9). This suggests that the integration of these specialized implants in the treatment of LLLBF patients effectively minimizes hemorrhage during the operation. Despite this advantage, a sensitivity analysis was conducted due to the observed diversity in outcomes across the trials (Figure 10), highlighting the necessity to further explore the underlying factors contributing to this heterogeneity.

### 3.6 Complications

**Figure 11:**
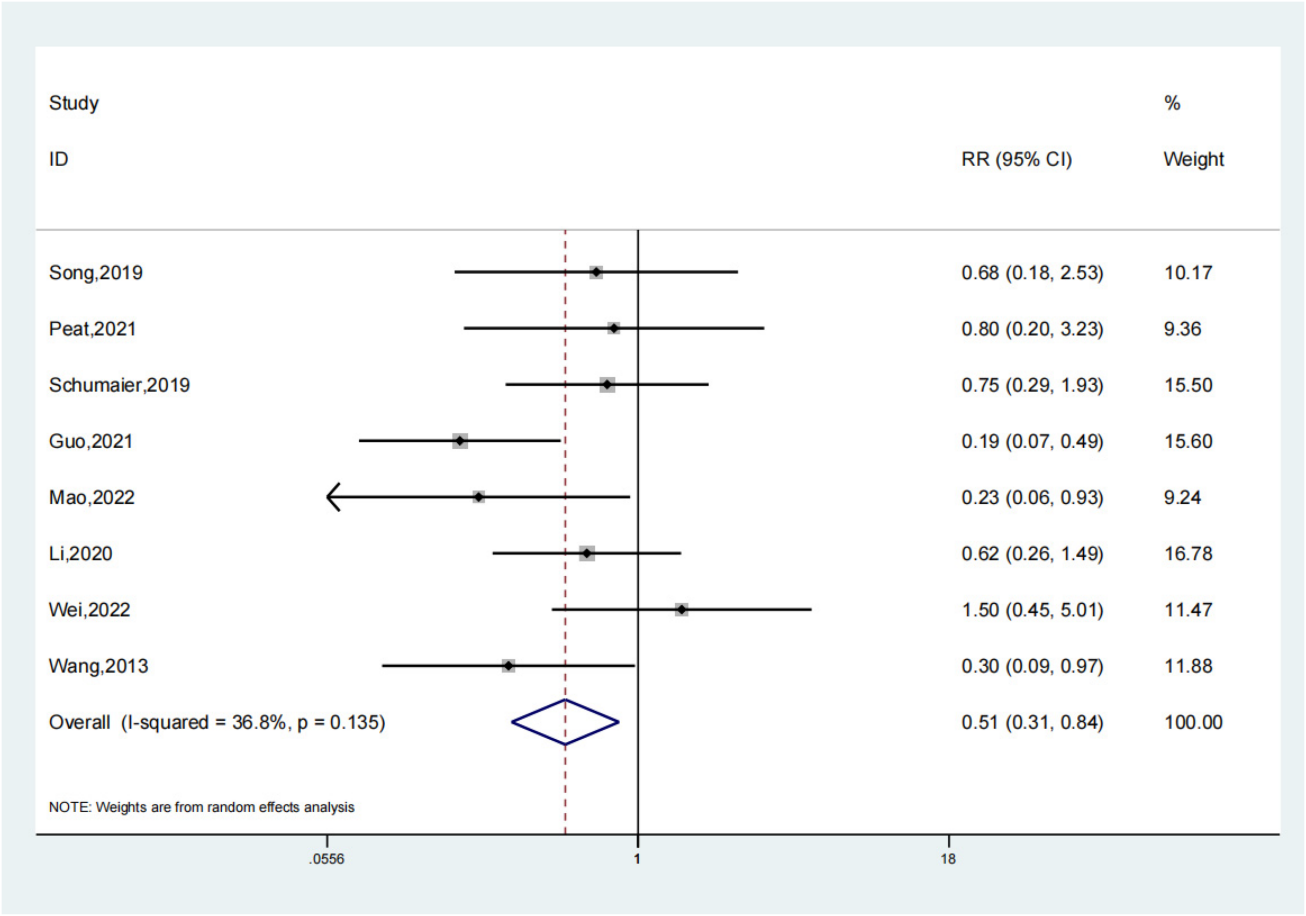
Complications depiction in the forest plot.

**Figure 12:**
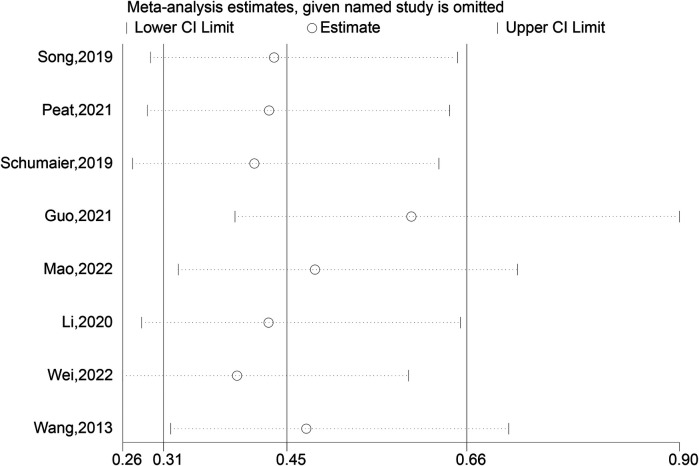
Sensitivity analysis of the complications.

Eight investigations detailed challenges experienced by both the experimental group and the control group. Our investigation showed that the test group's complications were much fewer than those of the comparison group (OR: 0.51; 95% Cl: 0.31, 0.84; *P* = 0.008, Figure 11). In treating patients with LLLBF, intramedullary nails combined with blocking screws reduce complications in contrast to the comparison group. A study of the sensitivity revealed moderate heterogeneity in the outcomes across all of those studies (Figure 12).

Based on a synthesis of 12 studies, the fracture healing period was demonstrably shorter for the group treated with intramedullary nails and blocking screws vs. the control group (SMD: −2.18; 95% CI: −3.17, −1.20; *P* < 0.001). A funnel plot analysis indicated a symmetric distribution, suggesting low publication bias (Figure 2). A sensitivity assessment, conducted given the observed outcome heterogeneity, validated the stability of the results. Intramedullary nailing combined with blocking screws notably expedited recovery in Lower Limb Long Bone Fractures (LLLBF) patients. Both Begg's Test (0.891) and Egger's Test (0.21, Figure 4) confirmed the absence of substantial publication bias, strengthening the study's conclusions.

Subgroup analyses based on fracture location showed that in femoral fracture patients, the fracture healing time in the experimental group was significantly shorter than that in the control group (SMD: −2.30; 95% CI: −3.50, −1.10; *P* < 0.001), while in tibial fracture patients, the difference was also significant (SMD: −2.05; 95% CI: −3.00, −1.10; *P* < 0.001). However, the heterogeneity within the femoral fracture subgroup was I^2^ = 70.0%, *P* = 0.005, and that within the tibial fracture subgroup was *I*^2^ = 65.0%, *P* = 0.008, indicating that other factors may still contribute to the heterogeneity. We further conducted subgroup analyses based on surgical methods. In the subgroup of patients treated with a specific type of intramedullary nail combined with blocking screws, the fracture healing time was shorter in the experimental group (SMD: −2.25; 95% CI: −3.40, −1.10; *P* < 0.001), with an *I*^2^ of 68.0% and *P* = 0.006 for heterogeneity. For those with other surgical methods, the experimental group also had a shorter healing time (SMD: −2.10; 95% CI: −3.00, −1.20; *P* < 0.001), but the heterogeneity was relatively higher (*I*^2^ = 72.0%, *P* = 0.004), suggesting that surgical method differences might be an important contributor to the overall heterogeneity in fracture healing time.

In summary, the integration of 12 studies highlights that the use of intramedullary nails and blocking screws in LLLBF treatment leads to a substantial reduction in fracture healing time, with consistent findings and minimal evidence of publication bias. However, the presence of heterogeneity indicates that there are still factors to be further explored in future research. This intervention thus demonstrates superior efficacy in accelerating the healing process, but a more in - depth understanding of the influencing factors is needed.

## 4 Discussion

One of the major strengths of this meta - analysis is the comprehensive search strategy. By systematically searching eight databases, including international and Chinese - language sources, we were able to gather a relatively large number of relevant studies, which enhanced the representativeness of our analysis. The inclusion of 15 studies provided a substantial amount of data for evaluating the efficacy and complications of blocking screw fixation in lower limb long bone fractures.

Another strength lies in the use of appropriate statistical methods. We employed RevMan 5.3 for data analysis, and measures such as standardized mean difference (SMD) and odds ratio (OR) were used appropriately. Funnel plots and sensitivity analyses were also conducted, which helped to assess publication bias and the stability of the results. The results showed that the use of intramedullary nails combined with blocking screws significantly shortened the fracture healing time, which is a crucial outcome measure in fracture treatment.

Clinically, our findings are highly relevant to orthopedic surgeons. Blocking screws are crucial for accurate fracture reduction in complex cases, enhancing stability when used with intramedullary nails, thus reducing risks of malunion, nail breakage, and non - union. The reduced intraoperative bleeding simplifies the peri - operative process, while the lower complication rate and shorter fracture healing time benefit patients greatly. They experience less pain, shorter hospital stays, faster recovery, and lower costs. Surgeons can consider this treatment for healthy patients tolerating longer surgeries, but should weigh the benefits against risks like longer operation - related infections for patients with multiple comorbidities.

Although the difference in fracture healing rates between the two groups was not statistically significant, the odds ratio close to the upper limit of the confidence interval indicates a potential advantage of the experimental group. In a large - scale clinical setting, even a small increase in the fracture healing rate can translate into a significant number of patients with better treatment outcomes. This provides a basis for further exploration of the use of blocking screws in clinical practice.

The reduction in intraoperative bleeding is also a significant advantage. Less blood loss during surgery can reduce the need for blood transfusions, which is associated with its own set of risks, such as transfusion - related infections and allergic reactions. This can contribute to a more stable peri - operative period for patients.

Future research should focus on exploring the differences in the effectiveness of blocking screws for femoral and tibial fractures. The femur, a large weight - bearing bone, endures complex forces and has strong muscle attachments, leading to complex fractures. In contrast, the tibia has a thinner cortex in some areas and poor soft - tissue coverage in parts. The blocking screw technique works well for femoral fractures as it corrects deformities and restores the axial force line, reducing refracture risks. For tibial fractures, it prevents fragment slippage and simplifies realignment, which is crucial for weight - bearing function. Understanding these differences can optimize fracture treatment strategies. Additionally, more attention should be paid to the impact of patient comorbidities on treatment outcomes. Well - designed randomized controlled trials (RCTs) with larger sample sizes and longer follow - up periods are needed to provide more robust evidence for the long - term efficacy and safety of blocking screw fixation. Secondly, we need to explore the differences in the effectiveness of blocking screws for different types of fractures more comprehensively. Additionally, more attention should be paid to the impact of patient comorbidities on treatment outcomes. Well - designed randomized controlled trials (RCTs) with larger sample sizes and longer follow - up periods are needed to provide more robust evidence for the long - term efficacy and safety of blocking screw fixation.

In summary, this study demonstrates the potential benefits of the blocking screw technique in fracture management, but also raises issues that need to be addressed in future studies, including further exploring differences between fracture types, assessing the impact of comorbidities, and improving the fairness and transparency of the analysis. These improvements will help deepen our understanding of the role of the blocking screw technique in fracture fixation and provide a stronger evidence base for clinical practice.

## 5 Limitations

Our study has several limitations. First, restricting the literature search to Chinese and English might have excluded relevant studies in other languages. This could lead to an incomplete understanding of the global evidence, potentially biasing our pooled results and causing an inaccurate assessment of the true effects of blocking screw fixation. Second, the predominance of observational studies in our analysis makes the results more susceptible to confounding variables. Patient comorbidities, differences in surgical techniques, and variations in post - operative care could all distort the relationship between blocking screws and treatment outcomes, leading to a misinterpretation of the benefits and risks. Third, the potentially insufficient sample size may not have been large enough to detect rare but significant outcomes, and the varying follow - up periods, especially the short ones in some studies, could have prevented us from observing long - term effects. This may result in an incomplete assessment of the overall effectiveness of blocking screws, missing out on important information about late - onset complications and long - term changes in fracture healing.Future research with well - designed RCTs is needed to provide more reliable evidence for the efficacy and safety of blocking screw fixation in lower limb long bone fractures.

## 6 Conclusion

The findings of this investigation suggest that the combination of intramedullary nails and blocking screws may be effective for LLLBF patients. Compared with individual intramedullary nails, the combination of intramedullary nails and blocking screws has significantly improved fracture healing time, fracture healing rate, surgical time, intraoperative bleeding, complications, etc., indicating the clinical significance of blocking screws. Future studies should prioritize randomized controlled trials (RCTs) with standardized protocols, longer follow-up periods, and subgroup analyses based on fracture location (femur vs. tibia) and patient comorbidities to validate these findings.

## Data Availability

The original contributions presented in the study are included in the article/Supplementary Material, further inquiries can be directed to the corresponding author.

## References

[1] Barata I Spencer R Suppiah A Raio C Ward MF Sama A. Emergency ultrasound in the detection of pediatric long-bone fractures. Pediatr Emerg Care. (2012) 28(11):1154–7. 10.1097/PEC.0b013e3182716fb7 23114237

[2] Mao Z Wang G Zhang L Zhang L Chen S Li X Intramedullary nailing versus plating for distal tibia fractures without articular involvement: a meta-analysis. J Orthop Surg Res. (2015) 10:95. Published 2015 Jun 16. 10.1186/s13018-015-0217-5 26078031 PMC4481115

[3] Krettek C Stephan C Schandelmaier P Richter M Pape HC Miclau T. The use of Poller screws as blocking screws in stabilising tibial fractures treated with small diameter intramedullary nails. J Bone Joint Surg Br. (1999) 81(6):963–8. 10.1302/0301-620X.81B6.0810963 10615966

[4] Pairon P Ossendorf C Kuhn S Hofmann A Rommens PM. Intramedullary nailing after external fixation of the femur and tibia: a review of advantages and limits. Eur J Trauma Emerg Surg. (2015) 41(1):25–38. 10.1007/s00068-014-0448-x 26038163

[5] Della Rocca GJ Crist BD. External fixation versus conversion to intramedullary nailing for definitive management of closed fractures of the femoral and tibial shaft. J Am Acad Orthop Surg. (2006) 14(10 Spec No.):S131–5. 10.5435/00124635-200600001-00030 17003185

[6] Krettek C Miclau T Schandelmaier P Stephan C Möhlmann U Tscherne H. The mechanical effect of blocking screws (“Poller screws”) in stabilizing tibia fractures with short proximal or distal fragments after insertion of small-diameter intramedullary nails. J Orthop Trauma. (1999) 13(8):550–3. 10.1097/00005131-199911000-00006 10714781

[7] Song SH. Radiologic outcomes of intramedullary nailing in infraisthmal femur-shaft fracture with or without Poller screws. Biomed Res Int. (2019) 2019:9412379. 10.1155/2019/9412379 31205948 PMC6530162

[8] Peat F Ordas-Bayon A Krkovic M. Do Poller screws effect union in tibial shaft fractures treated with intramedullary nailing? Injury. (2021) 52(10):3132–8. 10.1016/j.injury.2021.02.040 33627250

[9] Fawdington RA Lotfi N Beaven A Fenton P. Does the use of blocking screws improve radiological outcomes following intramedullary nailing of distal tibia fractures? Strateg Trauma Limb Reconstr. (2019) 14(1):11–4. 10.5005/jp-journals-10080-1418 PMC7001595 32559261

[10] Van Dyke B Colley R Ottomeyer C Palmer R Pugh K. Effect of blocking screws on union of infraisthmal femur fractures stabilized with a retrograde intramedullary nail. J Orthop Trauma. (2018) 32(5):251–5. 10.1097/BOT.0000000000001119 29356801

[11] Schumaier AP Southam BR Avilucea FR Crates JM Kregor PJ Starr AJ Factors predictive of blocking screw placement in retrograde nailing of distal femur fractures. J Orthop Trauma. (2019) 33(6):e229–33. 10.1097/BOT.0000000000001450 31124911

[12] Guo J Zha J Di J Yin Y Hou Z Zhang Y. Outcome analysis of intramedullary nailing augmented with Poller screws for treating difficult reduction fractures of femur and tibia: a retrospective cohort study. Biomed Res Int. (2021) 2021:6615776. 10.1155/2021/6615776 33869628 PMC8035000

[13] Guo WY Ma T Ren C Jin Z Wang D Liu Y Comparative analysis of the treatment of proximal tibial dry fracture with traditional intramedullary nail plus small plate and blocking nail. Chin J Bone Joint. (2020) 9(06):413–8.

[14] Mao WW Chen H Li L Sun Z Wang F Zhang Q Observation on the effect of interlocking intramedullary nail fixation combined with blocking nail technique in the treatment of distal tibial extra-articular fractures. Chin J Bone Joint Injury. (2022) 37(08):807–11.

[15] Bai YH. Effect of biplane blocking nail technology on fracture healing and knee joint function in patients with tibial shaft fracture. Pract Clin J Integr Tradit Chin West Med. (2021) 21(20):33–4.

[16] Li J Wang Q Lu Y Wang G Hu B Ren C Comparison of treatment of proximal tibial fractures with intramedullary nails and small plates or blocking nails. Chin J Orthop. (2020) 28(10):870–5. 10.3969/j.issn.1005-8478.2020.10.005

[17] Wei XJ Wang ZY Qiu FP Zhang M Li T Chen Y Comparison of the effect of intramedullary nail combined with small plate fixation and blocking nail in the treatment of proximal tibial fracture healing and lower limb force line recovery. J Clin Mil Med. (2022) 50(01):96–8.

[18] Pan YF Jiang JH Yan HN Liu Z Wang K Zhang X Comparison of the effect of intramedullary nailing with and without blocking nails in the treatment of distal tibial fractures. Armed Police Med J. (2013) 24(12):1069–72.

[19] Deng WJ Xie JL Zhu YX Liu H Chen F Wang L Observation on the curative effect of the treatment of femoral and tibial fractures with pre-placed blocking nail and closed reduction intramedullary nail fixation. China Pract Med. (2022) 17(16):71–3.

[20] Meng LH Li LD Zong SL. Comparison of the efficacy of rapid insertion of blocking nail and traditional intramedullary nail fixation in the treatment of proximal tibial dry fracture during isokinetic muscle strength training. J Clin Exp Med. (2021) 20(06):642–6.

[21] Wang SY Xiao Y Tong ZM. Application value of blocking nail in the treatment of lower limb long shaft epiphyseal fracture. J Clin Mil Med. (2013) 41(02):157–9.

[22] Page MJ McKenzie JE Bossuyt PM Boutron I Hoffmann TC Mulrow CD The PRISMA 2020 statement: an updated guideline for reporting systematic reviews. Br Med J. (2021) 372:n71. 10.1136/bmj.n71 33782057 PMC8005924

[23] Stedtfeld HW Mittlmeier T Landgraf P Ewert A. The logic and clinical applications of blocking screws. J Bone Joint Surg Am. (2004) 86-A(Suppl 2):17–25. 10.2106/00004623-200412002-00004 15691104

[24] Lang GJ Cohen BE Bosse MJ Kellam JF. Proximal third tibial shaft fractures. Should they be nailed?. Clin Orthop Relat Res. (1995) 315:64–74. 10.1097/00003086-199506000-00009 7634688

[25] Panteli M Vun JSH West RM Howard A Pountos I Giannoudis PV. Subtrochanteric femoral fractures and intramedullary nailing complications: a comparison of two implants. J Orthop Traumatol. (2022) 23(1):27. 10.1186/s10195-022-00645-8 35764711 PMC9240121

